# 

**Published:** 1885-01

**Authors:** 


WHOLE NUMBER, CCLXXII.

JANUARY, 1885.

NUMBER 6, VOLUME XXIV.

### THE BUFFALO Medical and Surgical Journal.

EDITORS:

THOS. LOTHROP, M. D., A. R. DAVIDSON, M. D.,

PUBLISHED BY THE MEDICAL JOURNAL ASSOCIATION.

No. 5 WEST CHIPPEWA ST.

Entered at the Post-Office at Buffalo, N. Y., as Second-class Mail Matter.

